# Non-invasive imaging of single human motor units

**DOI:** 10.1016/j.clinph.2020.02.004

**Published:** 2020-06

**Authors:** Matthew G. Birkbeck, Linda Heskamp, Ian S. Schofield, Andrew M. Blamire, Roger G. Whittaker

**Affiliations:** a Newcastle University Translational and Clinical Research Institute (NUTCRI), Newcastle University, Newcastle upon Tyne NE1 7RU, UK; b Newcastle Biomedical Research Centre, Newcastle University, Newcastle upon Tyne NE4 5PL, UK; c Northern Medical Physics and Clinical Engineering, Freeman Hospital, Newcastle upon Tyne NHS Foundation Trust, Newcastle upon Tyne NE7 7DN, UK

**Keywords:** CSA, Cross Sectional Area, EDL, Extensor Digitorum Longus, EMG, Electromyography, FOV, Field Of View, GL, Gastrocnemius Lateralis, GM, Gastrocnemius Medialis, MRI, Magnetic Resonance Imaging, MU, Motor Unit, MUMRI, Motor Unit Magnetic Resonance Imaging, PL, Peroneus Longus, ROI, Region Of Interest, SOL, Soleus, TA, Tibialis Anterior, TE, Echo Time, TR, Repetition Time, Diffusion weighted imaging, Motor unit, Alternation, Electromyography

## Abstract

**Objective:**

To determine the size, shape and distribution of single human motor units *in-vivo* in healthy controls of different ages.

**Methods:**

A novel diffusion-weighted magnetic resonance imaging (MRI) technique was used in combination with in-scanner electrical stimulation to quantify the shape, cross-sectional area, and dimensions of individual motor units in 10 healthy subjects.

**Results:**

Thirty-one discrete motor units were studied. The majority were elliptical or crescent shaped, but occasional split motor units were observed. The mean motor unit cross sectional area was 26.7 ± 11.2 mm^2^, the mean maximum dimension was 10.7 ± 3.3 mm, and the mean minimum dimension was 4.5 ± 1.2 mm. Subjects aged over 40 had significantly larger maximum dimensions than those below this age (p < 0.05).

**Conclusions:**

Motor unit MRI (MUMRI) is a novel technique capable of revealing the size, shape and position of multiple motor units in human muscles. It is reproducible, non-invasive, and sufficiently sensitive to detect physiologically relevant changes in motor unit morphology with age.

**Significance:**

To our knowledge, these results provide the first imaging assessment of human motor unit morphology. The technique shows promise both as a diagnostic tool and as a biomarker in longitudinal studies of disease progression.

## Highlights

• A novel MRI technique capable of detecting the size, shape and distribution of human motor units is described.
• Human motor units have a range of different outlines including elliptical, complex or split.
• This technique demonstrates a heterogeneous remodelling of motor units with age.

## 1 Introduction

The size of a motor unit is a critical determinant of its physiological action (McPhedran et al., 1965), and understanding changes in motor unit structure in the setting of neuromuscular diseases is of fundamental importance in the interpretation of diagnostic clinical electromyography (EMG) (Whittaker, 2012).

Much of our basic understanding of motor unit morphology arises from glycogen depletion experiments in animals and rarely in humans (Edström and Kugelberg, 1968, Garnett et al., 1979). These require the isolation and prolonged electrical stimulation of a single motor axon such that the glycogen stores in the muscle fibres that this axon innervates are selectively depleted.

In healthy cat muscles, these experiments have shown that the fibres in a single motor unit are widely separated, typically occupying an oval or circular territory covering between 8 to 76% of the total muscle cross-sectional area (CSA) (Bodine et al., 1988). Following incomplete section of a motor nerve, the surviving motor units in the supplied muscle show a higher fibre density (Kugelberg et al., 1970). These changes are usually interpreted as resulting from collateral sprouting from surviving motor axons into adjacent regions of denervated muscle (Thompson and Jansen, 1977).

Performing such technically challenging and time-consuming studies in humans is clearly impractical, and it remains a matter of conjecture as to whether the results obtained from rodents and cats using this technique are directly translatable to humans. For example, human motor units contain 2–4 times as many muscle fibres as cats (Buchthal and Schmalbruch, 1980). Nevertheless, the concept of motor unit remodelling in neurogenic pathologies is supported by several clinical neurophysiological techniques: the fibre density in re-innervated muscle is increased compared to healthy muscle compatible with increased collateral sprouting (Sandberg, 2014); the macro-EMG signal is increased, compatible with a greater total number of innervated muscle fibres per motor unit (Stålberg, 1982) and the corridor length of a single motor unit measured using scanning EMG is increased, consistent with it occupying a greater CSA (Gootzen et al., 1992). Similar, albeit less conspicuous, changes occur in ageing individuals in whom enlarged motor units are seen as a result of motor unit drop-out and compensatory re-innervation (Stålberg and Fawcett, 1982, Larsson, 2003, Piasecki et al., 2016).

However, it remains the case that none of these techniques directly measure motor unit size and shape; even multi-electrode and scanning EMG records only a single corridor which, depending on where this intersects with the motor unit, may or may not accurately reflect its size (Erminio et al., 1959, Buchthal et al., 1960, Stålberg and Eriksson, 1987). Scanning EMG also reveals ‘silent areas’ in which no electrical activity is seen (Stålberg and Dioszeghy, 1991). This challenges the model of human motor units as a single contiguous region, and raises the possibility either of a more complex outline or of discrete ‘subunits’.

We recently developed a novel imaging technique based on diffusion-weighted MRI which is sensitive to the contraction of skeletal motor units (Whittaker et al., 2019). We now apply this technique in conjunction with in-scanner electrical nerve stimulation to perform the first systematic study of human motor unit size and shape in healthy controls of differing ages.

## 2 Methods

### 2.1 Subjects and experimental set up

**Fig. 1:**
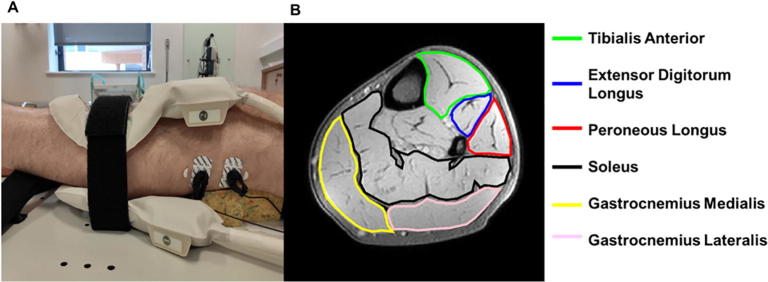
(A) Placement of the receive coil and the stimulation electrodes for fibular nerve stimulation. (B) High resolution anatomical axial Dixon image of the lower leg. Stimulation of the fibular nerve activates the tibialis anterior (green), extensor digitorum longus (blue) and peroneus longus (red). Stimulation of the tibial nerve activates the soleus (black), gastrocnemius medialis (yellow) and gastrocnemius lateralis (pink). (For interpretation of the references to colour in this figure legend, the reader is referred to the web version of this article.)

Ten healthy subjects (8 male, age = 26–84 years; 2 female, age = 30–32 years) were scanned using a 3 T Achieva X MR scanner (Philips Medical Systems, Best, The Netherlands). Subjects were included if they could lie flat in the scanner for up to 60 minutes, and were excluded if they had contra-indication to MRI scanning or a clinical history of neuromuscular disease. Subjects lay supine on the scanner bed and a pair of 10 cm elliptical flexible surface coils (FlexM Philips Medical Systems, Best, The Netherlands) were positioned above and below the lower leg muscles (Fig. 1A). The knee was supported such that the lower leg muscles were not compressed. A pair of stimulating electrodes (Cleartrace, ConMed, New York, USA) were placed over the left common fibular nerve or tibial nerve. These were connected to a programmable stimulator (DS5; Digitimer, Ft Lauderdale, Florida, USA) via MR compatible coaxial cables with low-pass filters (Minicircuits, New York, USA) at the Faraday cage.

### 2.2 MR data acquisition

Axial anatomical images were collected with a multi-echo Dixon sequence in each volunteer (field of view (FOV) = 160 × 160 mm, 1 × 1 mm in-plane resolution, 7.5 mm slice thickness, repetition time (TR) = 180 milliseconds (ms), echo times (TE) = 3.45, 4.6 and 5.75 ms). Axial motor unit scans were collected using a diffusion weighted spin-echo echoplanar imaging sequence with diffusion sensitization along the predominant muscle fibre axis (FOV = 160 × 160 mm, 1.5 × 1.5 mm in-plane resolution, 7.5 mm slice thickness, TR/TE = 1000/36 milliseconds, Δ/δ = 16.9/2.2 milliseconds, b-value = 20 s/mm^2^). The two slices were positioned on the thickest part of the calf. Scans were collected both during full muscle relaxation and during electrical stimulation. Electrical stimulation was performed at a frequency of 1 Hz for all experiments, with a bipolar square pulse wave 0.3 ms duration. The inter-electrode distance was 5 cm and cathode placed distal.

**Supplementary Fig. S1:**
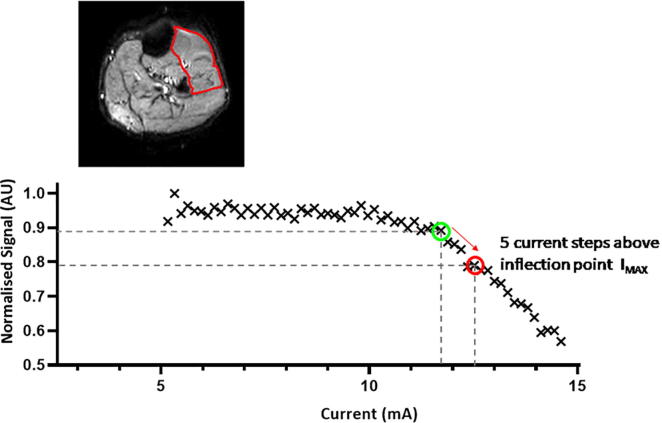
Typical example of the signal intensity in the muscles of the anterior compartment of the lower leg (delineated in red) during the coarse grain ramp up experiment. The green circle reflects the inflection point, i.e. current strength at which the signal intensity in the anterior compartment muscles starts to decrease (motor units become active). From this inflection point, starting current I_MAX_ for the fine-grain ramp down experiment is determined (red circle).

In order to characterise the morphology of individual motor units, it was essential that only a few units were active within the imaging slice. To achieve this, a dynamic scan (1 image acquired per second) was collected during which the stimulating current was increased in steps of 0.1–0.5 mA until a clear level of contrast was observed between the stimulated and non-stimulated muscles (60 dynamics, acquisition time: 1 min 3 s) (Supplementary Video A). Such a level of activation was typically achieved with stimulation currents of around 12 mA; we call this scan a coarse-grain scan. On this scan a region of interest (ROI) was delineated in the stimulated muscle, and a profile of current against signal intensity was produced (Supplementary Fig. S1). From this profile an inflection point was determined that defined the stimulation current at which activity was first visible.

A second scan was then collected with the stimulating current set to start at a level corresponding to five current steps higher than the inflection point (I_MAX_) (Supplementary Fig. S1). The stimulating current was then decreased in steps of 0.01 mA, each current level being repeated 5 times in order to observe motor unit alternation (van Dijk and Blok, 2008). This was repeated until no motor unit activity was observed (1080 dynamics, acquisition time: 18 min 3 s). We call this a fine-grain scan. To assess the reproducibility of the method, after the first fine-grain scan four of the ten volunteers were removed from the scanner bore, were asked to walk around the scanner with the stimulating electrodes still attached, placed back into the scanner bore and the fine-grain scan repeated.

### 2.3 Image analysis

The fine-grain images were masked using Fiji (Schindelin et al., 2012) by manually delineating the muscle(s) which were activated by the stimulated nerve to remove the non-activate muscles, blood vessels and background. For fibular nerve stimulation masked images contained the tibialis anterior (TA), extensor digitorum longus (EDL) and peroneus longus (PL) muscles; for tibial nerve stimulation masked images contained the gastrocnemius medialis and lateralis (GM, GL) and soleus (SOL) muscles (Fig. 1B). The use of extremely small current steps allowed us to observe probabilistic firing of the motor unit around its activation threshold (Dean et al. 2014). Critically this occurs in an all-or-none fashion, visible on the fine grain scans as flickering of spatially consistent regions of the image (see Supplementary Video B). Motor unit activity difference maps were created by selecting regions that displayed this alternating behaviour, as described below.

**Fig. 2:**
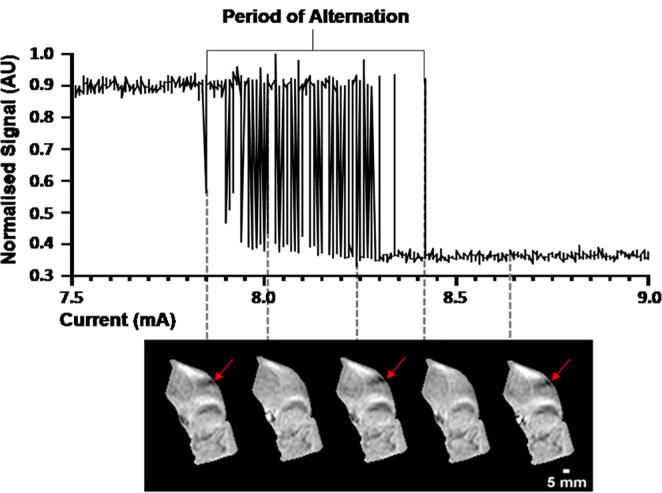
Signal intensity in a motor unit over time during the fine-grain experiment revealing the phenomena of alternation. If the current strength is slowly increased, at a certain current the signal intensity drops followed by a period where the signal intensity is alternatingly high or low (motor unit alternation), until the current reaches a level that the signal intensity remains low (continuously active motor unit). Panel of images correspond to different points during the period of alternation, showing an alternating unit indicated by the red arrows.

**Fig. 3:**
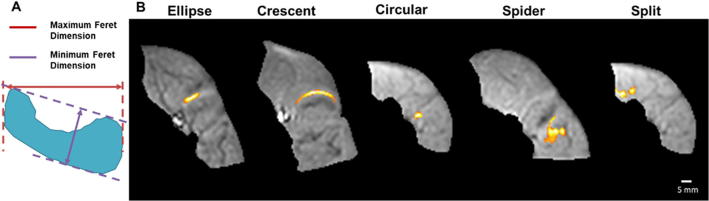
(A) Example motor unit shape indicating the maximum (red arrow) and minimum (purple arrow) Feret dimensions. (B) Typical examples of the five detected motor unit shapes. All examples are following fibular nerve stimulation and occur within the muscles of the anterior compartment of the leg. (For interpretation of the references to colour in this figure legend, the reader is referred to the web version of this article.)

**Supplementary Fig. S2:**
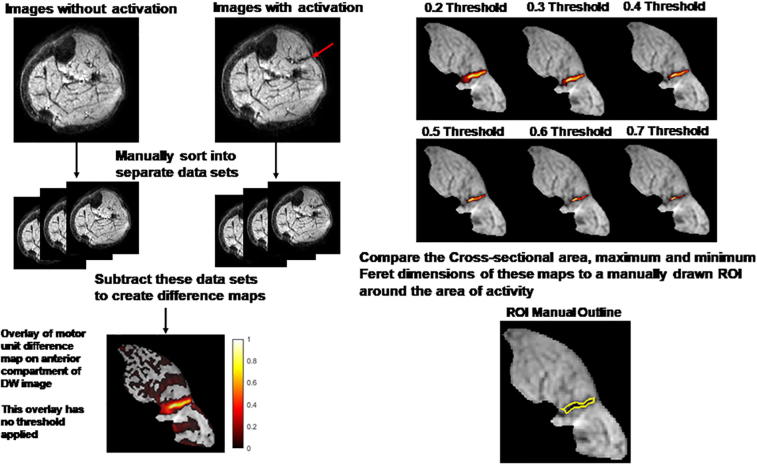
Process by which the 0.5 threshold was determined. Difference maps were created for each motor unit as discussed in Section 2.3. Difference maps were thresholded from 20 to 70% of normalised maximum pixel intensity. Motor unit metrics from thresholded images were compared with manually drawn regions of interest (ROIs), delineated by the two independent observers on original images of activity. The CSA, maximum and minimum Feret dimension from each thresholded map were then compared to those of the manually delineated region.

**Supplementary Fig. S3:**
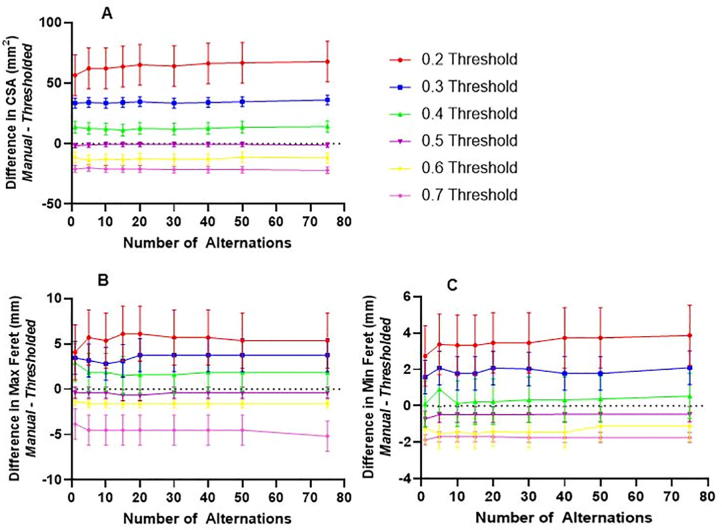
Example plot of different motor unit metrics. (A) CSA, (B) Maximum Feret dimension, (C) Minimum Feret dimension. Number of alternations (i.e. how many times one has selected yes when performing the first step in Supplementary Fig. S2), plotted against difference between the manually drawn ROI and the different threshold levels from Fig. 1. Plots show that the 0.5 threshold level is closest to zero difference for each metric. Furthermore plots show that the number of alternations selected does not influence the motor unit metric except for at very low number of alternations (e.g. 1, 5). For this reason all motor unit metrics reported are at 20 alternations. Twenty alternations was chosen as for one of the motor units analysed this was the maximum number of alternations selected.

For each area of activity we manually grouped dynamic images into images with and without signal voids (Fig. 2). These two groups of images were averaged and subtracted to reveal maps of motor unit activity. Due to the interdigitated nature of motor units, in subjects where more than one motor unit was activated, areas of activation were often very close to each other or overlapped. To remove interference from this nearby activity of other motor units, each map was thresholded. This was performed by normalising the map to the maximum signal intensity and then removing all voxels below a given percentage (Supplementary Fig. S2). A threshold of 0.5 was chosen, which was optimised as discussed in the Supplementary Material (Supplementary Figs. S2 & S3). CSA and maximum and minimum Feret dimensions (Fig. 3A) (defined as the distance between two parallel planes restricting the object) were calculated. All data were analysed offline by two independent observers, who developed the analysis protocol, using purpose-written scripts running in MATLAB 2019a (MathWorks, Natick, MA).

## 3 Statistics

Normality of the CSA and maximum and minimum Feret dimension data was assessed using the Shapiro-Wilk test. Comparison of metrics between muscles of the anterior compartment was performed using an ANOVA. Comparison of motor unit CSA and Feret dimensions between volunteers aged <40 years and >40 years respectively was performed using an unpaired two-tailed student’s t-test at 95% confidence level. An inter-observer comparison of the motor unit metrics was performed using a two way mixed intra-class correlation with absolute agreement and using a Bland-Altman analysis. All results are reported as mean ± standard deviation, unless otherwise stated.

## 4 Ethical Approval

This study in healthy volunteers was approved by the Newcastle University Ethics Committee (ref 1621/7484/2018). All subjects gave written informed consent prior to inclusion.

## 5 Results

### 5.1 Subjects and number of identified motor units

Of the ten subjects studied, seven had fibular nerve stimulation only, one had tibial nerve stimulation only and two underwent stimulation of both nerves. In all subjects, images showed evidence of motor unit activity. A total of 31 motor units were extracted from all subjects. The number of motor units detected per subject varied, with a median of 3 motor units (range: 1 to 7 motor units) detected per subject for fibular nerve stimulation and 1 motor unit (range: 1 to 2 motor units) for tibial nerve stimulation. (Table 1). The muscles in which we observed the most motor unit activity were the peroneus longus (11 motor units) and extensor digitorum longus (11 motor units) (Table 1).

**Table 1:** Participant demographics (ordered by age), what type of stimulation was given, which muscles were recruited and the number of units from each muscle. EDL – extensor digitorum longus, PL – peroneus longus, TA – tibialis anterior, SOL – soleus, GL – gastrocnemius lateralis and GM – gastrocnemius medialis.

Age	Sex	Fibular/tibial stimulation	Muscles Recruited	Number of units
26	M	Fibular	EDL;TA	1 EDL ; 1 TA
28	M	Fibular	EDL	2 EDL
29	M	Tibial	GM	2 GM
30	F	Both	EDL;PL;TA;GL	3 EDL; 2 PL; 2 TA ; 1 GL
32	F	Fibular	EDL;PL	1 EDL ; 3 PL
47	M	Both	TA;SOL	1 TA ; 1 SOL
52	M	Fibular	PL	3 PL
65	M	Fibular	EDL;TA	2 EDL ; 1 TA
80	M	Fibular	EDL; PL	1 EDL ; 3 PL
84	M	Fibular	EDL	1 EDL

### 5.2 Motor unit morphology

**Supplementary Fig. S4:**
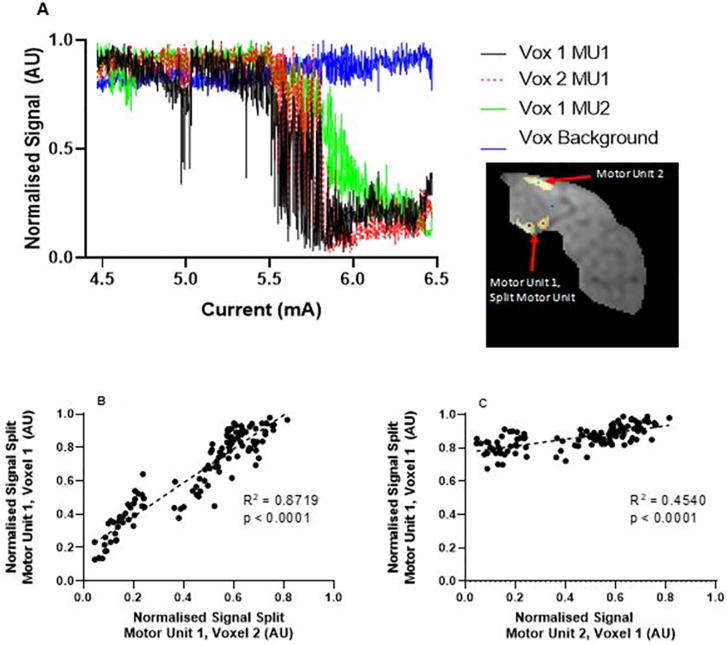
(A) Time series of the signal intensity in a split motor unit. The image shows the overlay of two motor units (1 split and one non-split). The graph shows the time series from the highlighted voxels in those motor units (Black – Voxel 1 split Motor Unit 1, Red – Voxel 2 split Motor Unit 1, Green – Voxel 1 Motor Unit 2, and Blue – Background voxel). (B-C) Correlation plots for: (B) Voxel 1 and 2 from within a split motor unit, (C) Voxel 1 from split motor unit against voxel 1 from motor unit 2.

The observed motor units were of different shapes, which we classified into five groups: elliptical, crescent, circular, spider and split (into two or more parts) (Fig. 3). The most common shape was elliptical (19 out of 31, see Table 2), occurring in four of the six different muscles studied. The next most common shape was crescent (5 out of 31), occurring in three of the muscles studied. Notably we observed two motor units which were split i.e. contained two or more separate regions which activated together. These were observed in two different volunteers in the tibialis anterior and in the soleus respectively. The border-to-border distance between the two spatially distinct regions was 1.5 mm for the motor unit in the tibialis anterior and 4.3 mm for the motor unit in the soleus. Analyses of time series data from the period of alternation for the split motor unit in volunteer 5, demonstrated a high correlation (r^2^ = 0.87) between voxels from areas of the split motor unit. Comparing time series from these voxels to a voxel from another motor unit showed a weaker correlation (r^2^ = 0.45) (Supplementary Fig. S4).

**Table 2:** Classification of motor unit shapes. EDL – extensor digitorum longus, PL – peroneus longus, TA – tibialis anterior, SOL – soleus, GL – gastrocnemius lateralis and GM – gastrocnemius medialis.

Descriptor	Example shape	Number	muscles
Ellipse		19	7 EDL; 6 PL; 4 TA2 GM
Crescent		5	2 EDL; 2 PL; 1 TA
Circular		3	2 PL; 1 GL
Spider		2	1 PL; 1 TA
Split (Into two or more parts)		2	1 TA; 1 SOL

### 5.3 Inter-observer comparison and reproducibility

**Supplementary Fig. S5:**
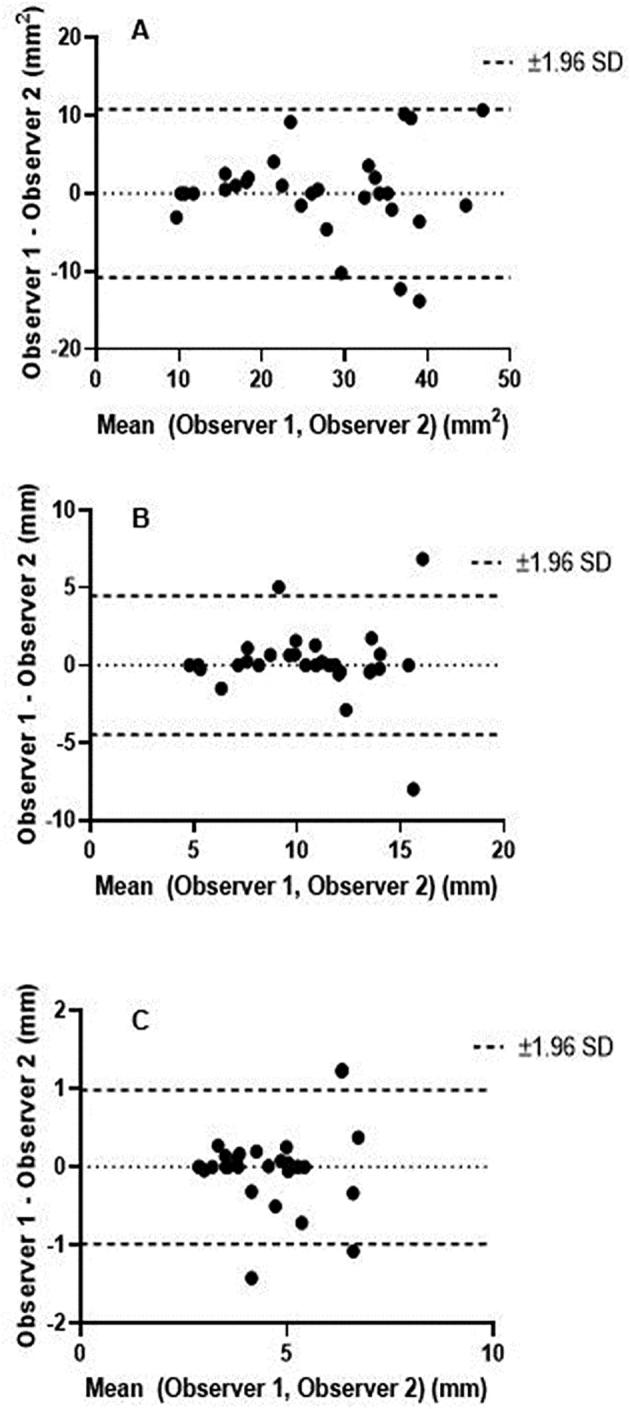
Bland Altman plots showing comparison between observers for each motor unit metric. (A) CSA, (B) Maximum Feret dimension, (C) Minimum Feret dimension.

All 31 motor units were analysed by two independent observers. Intra-class correlation coefficients (ICC) for each motor unit metrics were: ICC_CSA_ = 0.935, ICC_FeretMax_ = 0.868 and ICC_FeretMin_ = 0.957. Furthermore, motor unit metrics had a bias and coefficient of repeatability of 0.18 mm^2^ and 10.98 mm^2^ for CSA, 0.20 mm and 4.48 mm for maximal Feret diameter and −0.01 mm and 0.99 for minimal Feret diameter (Supplementary Fig. S5).

Four subjects underwent the fine-grain scan twice to test the repeatability of our imaging technique. All the motor units detected in the first scan were again detected on the second scan. The average absolute difference between the two scans for each observer was: for CSA (Observer 1 = 6.6 mm, Observer 2 = 6.8 mm); for the maximal Feret diameter (Observer 1 = 1.6 mm, Observer 2 = 1.8 mm); and minimal Feret diameter (Observer 1 = 0.7 mm, Observer 2 = 1.2 mm). In the case of CSA, this is the order of three acquisitions voxels (1.5 × 1.5 mm each voxel), and for the Feret dimensions is in the order of 1 acquisition voxel.

### 5.4 Motor unit cross sectional area & Feret dimensions

**Fig. 4:**
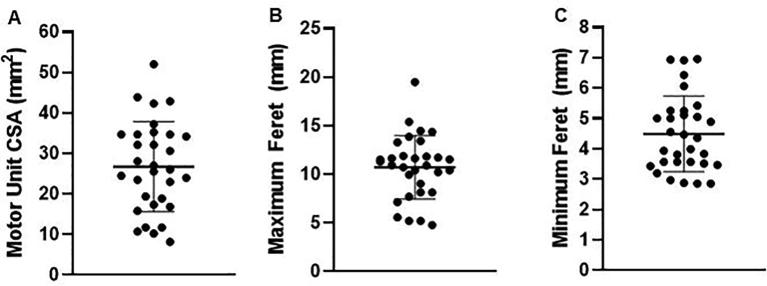
Scatter plot of the motor unit metrics for all of the observed motor units (n = 31). (A) Cross sectional area (CSA), (B) Maximum Feret dimension, (C) Minimum Feret dimension.

Motor unit metrics were normally distributed (Shapiro-Wilk statistics: W_CSA_ = 0.97; W_FeretMax_ = 0.96; W_FeretMin_ = 0.92). The average motor unit CSA was 26.7 ± 11.2 mm^2^, average maximum and minimum Feret diameters were 10.7 ± 3.3 mm and 4.5 ± 1.2 mm respectively (Fig. 4).

### 5.5 Comparison of motor unit metrics between muscles

**Fig. 5:**
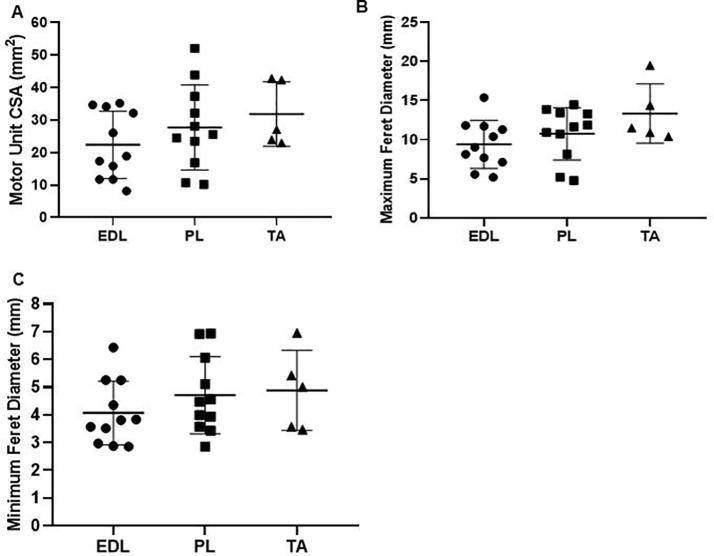
Motor unit metrics subdivided by muscle for the anterior compartment. (A) Cross sectional area (CSA). (B) Maximum Feret dimension. (C) Minimum Feret dimension. EDL – extensor digitorum longus, PL – peroneus longus, TA – tibialis anterior.

Since 28 of the 31 motor units were observed in the anterior compartment of the lower leg, we only compared the motor unit metrics between the extensor digitorum longus, peroneus longus and tibialis anterior. No significant difference was observed between these muscles for any motor unit metric (Fig. 5) (EDL *vs.* TA: p_CSA_ = 0.296, p_FeretMax_ = 0.091, p_FeretMin_ = 0.488; EDL *vs.* PL: p_CSA_ = 0.531, p_FeretMax_ = 0.608, p_FeretMin_ = 0.490; PL *vs.* TA: p_CSA_ = 0.784, p_FeretMax_ = 0.335, p_FeretMin_ = 0.968).

### 5.6 Change in motor unit metrics with age

**Fig. 6:**
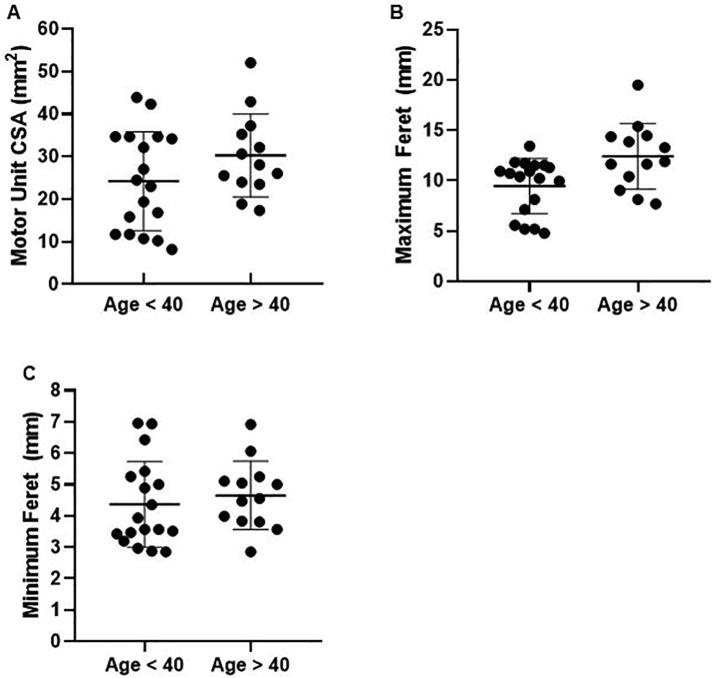
Effect of age on motor unit metrics. Pooled data from 5 subjects below 40 years of age (n = 18 motor units) and 5 subjects above 40 years of age (n = 13 motor units). (A) Cross sectional area (CSA). (B) Maximum Feret dimension. (C) Minimum Feret dimension.

**Supplementary Fig. S6:**
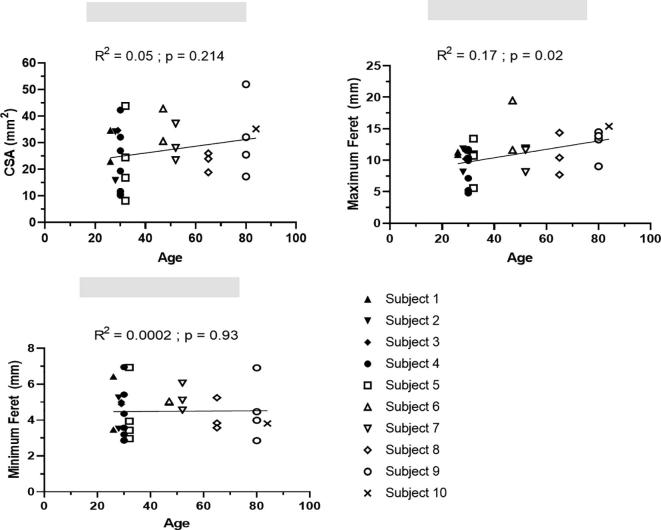
Correlation graphs between age and motor unit metrics. (A) Cross-sectional area (CSA), (B) Maximum Feret dimension, (C) Minimum Feret dimension.

The median age of volunteers was 39.5 years old. Therefore, we chose to separate volunteers into two groups <40 years and >40 years old. Subjects older than 40 years had a significantly larger maximum Feret dimension 12.4 ± 3.3 mm, compared to volunteers younger than 40 years old 9.5 ± 2.7 mm, p = 0.011 (Fig. 6). No difference was observed in the CSA and the minimum Feret dimension (p = 0.138 and p = 0.541 respectively). Furthermore, age correlated significantly with the maximum Feret dimension, and not with CSA and minimum Feret dimension (Supplementary Fig. S6). Three of the four motor units with a split or spider-shaped outline occurred in subjects over the age of 40.

### 5.7 Alternation characteristics of individual motor units

**Supplementary Fig. S7:**
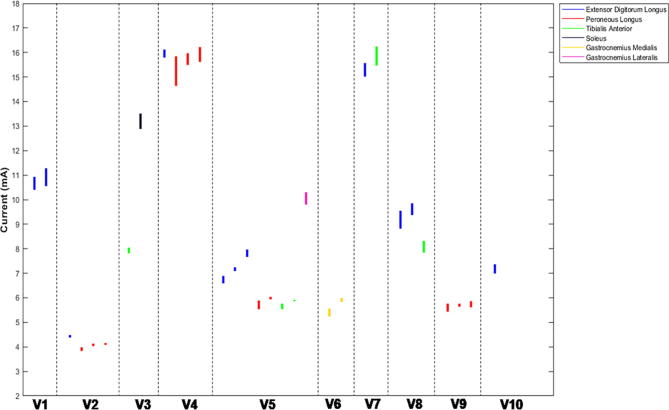
The current range for which alternation is observed displayed for each motor unit in each volunteers (V1–V10). Muscles indicated via colours (Extensor Digitorum Longus – Blue, Peroneous Longus – Red, Tibialis Anterior – Green, Soleus – Black, Gastrocnemius Lateralis– Pink, Gastrocnemius Medialis – Yellow).

The mean current range over which a motor unit exhibited alternation was 0.38 ± 0.26 mA, and the maximum and minimum current ranges were 1.20 mA and 0.05 mA respectively (Supplementary Fig. S7). The threshold current at which a motor unit first became active positively correlated with the period of alternation for that motor unit (p < 0.001; r^2^ = 0.537).

## 6 Discussion

Motor unit MRI (MUMRI) is a novel technique that allows the size, shape and distribution of multiple human motor units to be determined. Previously, this spatial information has only been obtainable in animal models using the glycogen depletion technique, and we believe this to be the first time that direct visualisation of motor unit outlines has been possible in humans.

In our sample of healthy controls, the maximum and minimum motor unit dimensions were 10.7 ± 3.3 mm and 4.5 ± 1.2 mm respectively. These are remarkably similar to results obtained using scanning electromyography (10–3 mm) (Stålberg and Dioszeghy 1991). However, in contrast to scanning EMG, up to 8 motor units can be detected simultaneously using MUMRI, the technique is entirely non-invasive, and rather than revealing activity from a single corridor it produces a 2D image of the entire motor unit cross-section.

The majority of the motor units had an elliptical or crescent-shaped outline, which agrees with data on motor unit outline from glycogen depletion experiments. We never observed motor units which spanned more than one muscle. Interestingly, we observed two ‘split’ motor units; that is areas of activity that alternated at the same time as each other and were spatially distinct from each other. We also detected motor units with a complex spider-shaped outline, with regions of activity interspersed with regions of no activity. ‘Silent zones’, where the motor unit potential drops to <50 µV p-p amplitude, have been observed in scanning EMG transects (Stålberg and Dioszeghy, 1991). It may be that these occur when the recording corridor transects a ‘split’ or spider-shaped motor unit, suggesting that human motor units may have a more complex anatomy than those in lower animals. We cannot say whether these seemingly discrete regions coalesce to form a contiguous structure further along the muscle. These questions could be further addressed by acquiring multiple imaging slices along the muscle or coronal/sagittal imaging.

We were careful to ensure that the observed signal voids represented individual motor units. We therefore limited our ‘fine-grain’ stimulation to cover a current range in which only the first few motor units were active and only analysed regions in which we observed clear motor unit alternation (van Dijk and Blok, 2008). Similarly, when analysing the second unit in a given muscle we deliberately chose a region which was spatially distant from the first analysed unit, and so on for subsequent units. This gives us confidence that the observed signal voids represent the activity of single motor units, but meant that regions containing overlapping motor units were excluded from our analysis. This highly conservative approach revealed a mean of 3 motor units per subject, though in one subject were able to analyse up to 8 motor units, because all motor units appeared spatially distinct from each other. Future work is needed to develop an analysis pathway capable of delineating overlapping motor units, and we are exploring methods using pixel-wise cross-correlation of active regions in an effort to increase the yield of the technique. Similarly, by limiting ourselves to low stimulation currents at which only a small number of motor units were active, we inevitably bias the results towards larger motor units which are preferentially recruited by electrical stimulation (Singh et al., 2000, Grill, 2015). One solution is to use voluntary activity to allow recruitment of smaller motor units and to remove the need for in-scanner electrical stimulation, and we are currently developing analysis methods to permit this.

We found the fibular nerve to be an easier site to study than the tibial nerve for two reasons; first, the current needed to achieve comparable levels of motor unit stimulation was lower in the fibular nerve, making it more comfortable for subjects. Second; the current range between stimulation of the first motor unit and supramaximal stimulation was larger for the tibial nerve (~8 mA) than the fibular nerve (~5 mA). This meant that for a given current range used in the fine gran scans, a larger number of motor units was detected with fibular nerve stimulation compared to tibial nerve stimulation. Both these factors presumably relate to the greater depth of the tibial nerve in the popliteal fossa. Although it is possible to increase the number of detected motor units in muscles innervated by deeper nerves, this is at the expense of increased stimulation intensities and longer scan times compared to more superficial stimulation sites.

Our ‘fine grain’ scans used a highly conservative current step of only 0.01 mA which meant they took a total of 18 minutes to perform. However, we found that the minimum current range over which alternation occurred was 0.05 mA, suggesting that we could increase the current steps by a factor of five whilst still ensuring the recruitment of single units with each current step. This could potentially reduce the scan time to just over 3 minutes, a duration that is feasible for clinical application. Further work is also needed to improve the image analysis techniques to allow motor units activated at higher stimulating currents and during voluntary contractions to be analysed in order to reveal activity in the whole motor unit pool.

Motor units interdigitate with several others, the fibres of any given unit making up only ~10% of a given cross sectional area (Brandstater and Lambert, 1973). This raises the question as to why activity in an individual motor unit should produce such a profound reduction in signal across multiple pixels. Adjacent motor units are closely mechanically coupled (Fritz et al., 1992) and it is likely that the area of signal drop-out reflects the contraction of one motor unit which then ‘pulls’ the adjacent (inactive) muscle fibres along with it. As such, the region in which movement occurs probably over-estimates the dimensions of the active motor unit. Conversely, it is likely that there are pixels at the periphery of the signal void which contain too few fibres from the motor unit in question to produce a detectable signal, under-estimating the true size of the motor unit. The net effect of these errors is unknown, and would require *in vitro* animal studies combining motor unit imaging with a glycogen depletion study to answer it definitively, but the concordance with scanning EMG suggest that it is relatively small.

We were interested to see whether MUMRI could detect physiologically relevant differences in motor unit morphology. It is known that as individuals’ age there is loss of motor units, compensatory re-innervation, and an increase in the size of surviving motor units (Larsson, 2003, Piasecki et al., 2016). Even in this relatively small sample size we were able to detect a statistically significant difference in maximum motor unit dimension in subjects aged over 40 compared to those aged under 40. We did not observe a corresponding change in the CSA or minimum Feret diameter between the two age groups, suggesting that age-related motor unit re-modelling is non-uniform, potentially resulting in complex motor unit outlines such as we observed. This is supported by the observation that the more complex motor unit outlines tended to occur in older subjects. If we assume that age-related changes in motor unit territory are a result of gradual denervation and re-innervation, these results appear to be at odds with the glycogen depletion studies of Kugelberg and Edstrom in rodents, in which re-innervation of a motor unit occurred only within the borders of the original unit (Kugelberg et al., 1970). More recent studies using scanning electromyography do however show significant increases in the corridor length of human motor units following re-innervation, implying an increased cross-sectional area of the unit (Stålberg and Dioszeghy, 1991). Interestingly this difference was seen in the tibialis anterior muscle but not in biceps brachii, and it may be that the changes that we observed in the leg muscles are not a general phenomenon. It is also possible that the observed differences arise as a result of age-related changes in the extracellular matrix, which in turn alters the degree to which the contraction of individual motor units deforms the surrounding muscle fibres, rather than any change in the territory of the motor unit itself. Our study also contained only 2 female subjects neither of whom were in the older age range, and further investigation is warranted to study the effect of sex and ageing on motor unit morphology.

MUMRI appears to be highly reproducible within a scanning session, with an average difference of three voxels for CSA and one acquisition voxel for Feret dimensions between acquisitions. Furthermore, the intra-class correlation coefficients were excellent and observers had a low inter-observer bias, demonstrating a robust analysis pipeline for this study. This, along with the non-invasive and well-tolerated nature of the technique, suggests potential for these metrics as imaging biomarkers in longitudinal studies. We are about to embark on such studies in patients with ALS, spinal muscular atrophy and sarcopenia.

In summary MUMRI is a robust and reproducible imaging technique which for the first time has allowed us to non-invasively study and quantify the size, shape and distribution of single human motor units *in-vivo*. Our findings show that the dimensions of motor units detected using our technique agree with those from the literature and the size of human motor units increases heterogeneously with normal aging. The metrics extracted from MUMRI data could be used as potential imaging biomarkers to distinguish between healthy and pathological muscle and follow disease progression over time. Further work is needed to validate the technique against conventional electrophysiological techniques in patients with neuromuscular diseases such as ALS and sarcopenia, and at the moment the yield of motor units is relatively low. However, with improvements in analysis techniques and the ability to image voluntary motor unit activation, we feel that MUMRI shows promise as a clinical tool alongside existing electrophysiological techniques.

## Declaration of Competing Interest

The authors declare that they have no known competing financial interests or personal relationships that could have appeared to influence the work reported in this paper.

## Appendix A Supplementary material

Supplementary video A Coarse grain scan from one subject. Current range for this scan (5.16–14.6 mA, total 60 dynamics). Anterior compartment of leg begins to show MU activity at 10.4 mA (around 4 s) and demonstrates progressive reduction in signal due to sequential motor unit activation.

Supplementary video B Section of fine grain scan from same subject as video A. Current range for this scan (10.05–12.2 mA, total 1080 dynamics). Clear alternation of a motor unit in extensor digitorum longus (EDL) muscle can be seen (from 2 s to 39 s). A second motor unit in the EDL can be observed to begin alternating at 13 s.
